# Characterization of microRNA and gene expression in the cochlea of an echolocating bat (*Rhinolophus affinis*)

**DOI:** 10.1002/ece3.9025

**Published:** 2022-06-22

**Authors:** Qianqian Li, Wenli Chen, Xiuguang Mao

**Affiliations:** ^1^ School of Ecological and Environmental Sciences, Institute of Eco‐Chongming (IEC) East China Normal University Shanghai China

**Keywords:** echolocation, horseshoe bats, microRNA, phenotypic variation, RNA‐seq

## Abstract

MicroRNAs (miRNAs) are important post‐transcriptional regulators of gene expression and play key roles in many biological processes, such as development and response to multiple stresses. However, little is known about their roles in generating novel phenotypes and phenotypic variation during the course of animal evolution. Here, we, for the first time, characterized the miRNAs of the cochlea in an echolocating bat (*Rhinolophus affinis*). We sampled eight individuals from two *R. affinis* subspecies with significant echolocation call frequency differences. We identified 365 miRNAs and 121 of them were novel. By searching sequences of these miRNAs precursors in multiple high‐quality mammal genomes, we found one specific miRNA shared by all echolocating bats but not present in all other nonecholocating mammals. The targeted genes of this miRNA included several known hearing genes (e.g., *KCNQ4* and *GJB6*). Together with the matched mRNA‐seq data, we identified 1766 differentially expressed genes (DEGs) between the two subspecies and 555 of them were negatively regulated by differentially expressed miRNAs (DEMs). We found that almost half of known hearing genes in the list of all DEGs were regulated negatively by DEMs, suggesting an important role of miRNAs in call frequency variation of the two subspecies. These targeted DEGs included several important hearing genes (e.g., *Piezo1*, *Piezo2,* and *CDH23*) that have been shown to be important in ultrasonic hearing of echolocating mammals.

## Graphical abstract

Characterization of microRNAs of cochlea in an echolocating bats. Identification of candidate miRNAs associated with ultrasonic hearing. Identification of miRNAs and their targets involved in ultrasonic hearing variation. Support the role of miRNAs in the origin of novel trait and phenotypic variation.

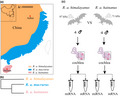

## 1 INTRODUCTION

MicroRNAs (miRNAs) are small noncoding RNAs that are approximately 22 nucleotide (nt) long and can mediate post‐transcriptional regulation of gene expression in multicellular eukaryotes (Bartel, 2009). In animals, mature miRNAs usually negatively regulate gene expression by inhibiting mRNA production via binding to the 3′ untranslated region (3′UTRs) of mRNA (Bartel, 2009). MiRNAs are well known for their vital roles in development (Bartel, 2004). During animal evolution, changes in miRNA repertoires have been linked to complexity (Berezikov, 2011; Heimberg et al., 2008; Hertel & Stadler, 2015). Evolution of new miRNAs is sometimes coincided with the origin of novel phenotypes, such as the cortical development in primates (Kosik & Nowakowski, 2018). More studies in other wild groups are needed to test whether miRNAs play important roles in generating novel traits.

Gene expression regulation has been considered as a key mechanism underlying phenotypic variations (Carroll, 2008; Jacob & Monod, 1961; Mank, 2017). Thus, miRNAs, as important post‐transcriptional regulator of gene expression, may also play important roles in phenotypic evolution of wild organisms. Up to now, few such studies have been conducted, such as in rapid phenotypic diversification of the Midas cichlids (Franchini et al., 2016, 2019).

Bats have multiple novel traits, such as flight, echolocation, and extreme longevity (Huang et al., 2016). Recently, a number of novel miRNAs in bats has been identified to have potential contributions to their extreme longevity (Huang et al., 2016, 2019). However, little is known about the role of miRNAs in echolocation. Bats use echolocation to explore environments and detect prey (Schnitzler et al., 2003). Horseshoe bats (Rhinolophidae) in particular have a specialized echolocation system. They emit long constant frequency components in their echolocation calls with the most energy contained in the second harmonic of the pulses (Siemers et al., 2005). These bats have auditory foveae responsible for response to frequencies of echolocation calls (Schuller & Pollak, 1979). When stationary, they omit calls at frequencies (resting frequency) that match to the frequencies of their hearing system (i.e., acoustic foveae) (Jones & Siemers, 2011). Thus, the resting frequency of horseshoe bats' echolocation calls can be considered as a morphological parameter associated with their acoustic foveae (Siemers et al., 2005). Considerable variations in peak frequencies of echolocation calls have been found among different horseshoe bats (Zhang et al., 2009) and also among different subspecies or geographic populations of several horseshoe bats (Jacobs et al., 2017; Sun et al., 2013). Using RNA‐seq data of cochlea tissue, the genetic basis of intraspecific variation in echolocation call frequency has been investigated among three *R. ferrumequinum* geographic populations (Zhao et al., 2019) and among three recently diverged subspecies of *R. affinis* (Sun et al., 2020). These two studies supported important roles of gene expression changes in intraspecific call frequency variations. However, little is known about the role of miRNAs in echolocation call frequency variation.

In this study, we focused on our previous study system, *R. affinis*. This species has three subspecies in China which diverged recently, <1 million years ago (Mao & Rossiter, 2020). Two of them (*R. a. himalayanus* and *R. a. macrurus*) are parapatric in the eastern region of mainland China and the third is from Hainan Island (*R. a. hainanus*). At intraspecific level *R. affinis* exhibits almost most divergent echolocation call frequency variation compared to other bat species although there are no significant differences in body size (e.g., forearm length) among the three subspecies (Mao et al., 2014). Specifically, *R. a. hainanus* and *R. a. macrurus* have similar echolocation call frequencies (71–74 kHz), consistent with the close phylogenetic relationships between them (Mao et al., 2010, 2013), whereas *R. a. himalayanus* shows over 15 kHz higher call frequency than each of them (Mao et al., 2010, 2013, 2014). Our previous phylogenetic and phylogenomic studies have detected extensive introgression of mtDNA between *R. a. himalayanus* and *R. a. macrurus* in their secondary contact region (Mao et al., 2013; Mao & Rossiter, 2020). Thus, we chose two allopatric subspecies (*R. a. himalayanus* and *R. a. hainanus*) as the system to assess the role of miRNAs during the divergence of echolocation call frequency to reduce the effect of introgression on patterns of gene expression (Dannemann et al., 2017; McCoy et al., 2017).

Here by characterizing miRNAs and mRNAs in the cochlea tissue from two subspecies of an echolocating bat (*R. a. himalayanus* and *R. a. hainanus*), we test the following two specific hypotheses. First, we hypothesize that there might be specific miRNAs which are essential in the evolution of echolocation. If the answer is yes, we would expect to identify shared miRNAs in all echolocating bats or echolocating mammals. Second, we hypothesize that miRNAs might be involved in regulating echolocation call frequency variation of the two subspecies. Following this hypothesis, we would expect to see multiple known hearing genes in the list of differentially expressed genes that are regulated by differentially expressed miRNAs identified between the two subspecies.

## 2 MATERIALS AND METHODS

### 2.1 Sampling

**FIGURE 1:**
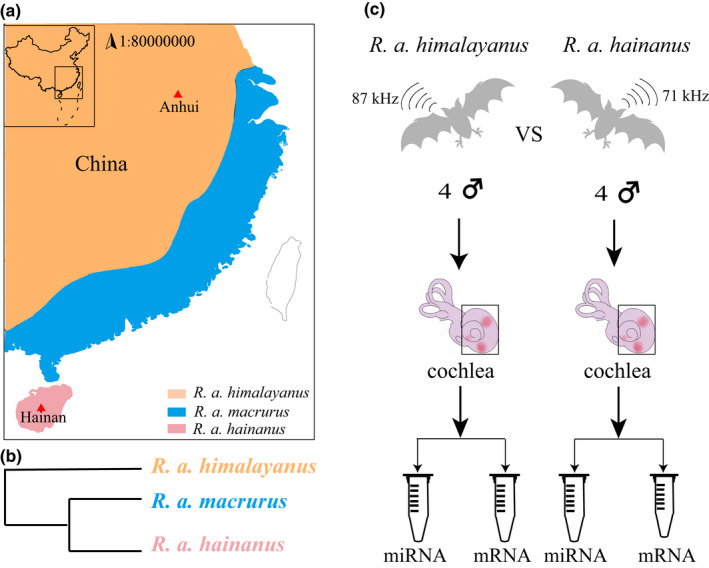
Sampling and experimental design. (a) The distribution of three subspecies of *R. affinis* in China. The red triangles indicate the sampling location in this study. (b) Phylogenetic relationships of three subspecies of *R. affinis* (modified from Sun et al., 2020). (c) Experimental design. Four individuals were sampled from each of the two subspecies with different echolocation call frequencies. Cochleae were used to generate miRNAs and mRNAs sequencing data

In this study, we captured eight adult males of *Rhinolophus affinis* including four *R. a. himalayanus* from Anhui province and four *R. a. hainanus* from Hainan Island (Table 1 and Figure 1). All bats were caught using mist nets when they fly out at dusk. Because it is difficult to determine the exact age of each bat, we roughly separated bats into adults and nonadults by checking for the joint of the fifth finger which remains swollen in juveniles and becomes knobbly in adults. In this study, only adult bats were used. For each bat, the resting frequency of echolocation calls was recorded using Avisoft UltraSoundGate 116Hnb kit (Avisoft, Berlin) and analyzed using BatSound (Fast Fourier Transformation size 1024, Hanning window). Bats were euthanized by cervical dislocation. For each bat, two inner ears were dissected and their vestibular portions were removed. The cochleae were then collected with RNase‐free tubes. Tissues were frozen immediately in liquid nitrogen and stored in a −80°C freezer. Our sampling procedure was approved by the National Animal Research Authority, East China Normal University (approval ID bf20190301).

**TABLE 1:** Detailed information about samples used in this study

Sample ID	Taxon	Sex	Call frequency (kHz)	Locality
Hai‐05	*hainanus*	Male	71.0	Hainan, China
Hai‐16	*hainanus*	Male	71.4	Hainan, China
Hai‐19	*hainanus*	Male	71.8	Hainan, China
Hai‐20	*hainanus*	Male	70.5	Hainan, China
Him‐12	*himalayanus*	Male	87.5	Anhui, China
Him‐13	*himalayanus*	Male	87.4	Anhui, China
Him‐35	*himalayanus*	Male	87.4	Anhui, China
Him‐36	*himalayanus*	Male	87.6	Anhui, China

### 2.2 RNA extraction, library construction, and sequencing

Total RNA from one cochlea of each individual was extracted with TRIzol (Life Technologies Corp., Carlsbad, CA, USA). The concentration and RNA integrity number (RIN) were determined using the Agilent 2100 Bioanalyzer system. The total RNA yields of samples were between 3.5 and 6.4 ug, and all samples had RIN values above seven except for one sample (Dryad file T1). Purified RNA from each sample was used for sequencing library construction. The mRNA and small RNA libraries were created with Illunima's TruSeq mRNA standard library preparation kit and NEBNext® Multiplex Small RNA Library Prep Set for Illumina® (NEB, USA), respectively. All libraries were quantified and qualified with Agilent 2100 Bioanalyzer and sequenced on Illumina Hiseq 2500/2000 for miRNA (single‐end 50 bp) and Novaseq 6000 for mRNA (pair‐end 150 bp).

### 2.3 MiRNA discovery

**FIGURE 2:**
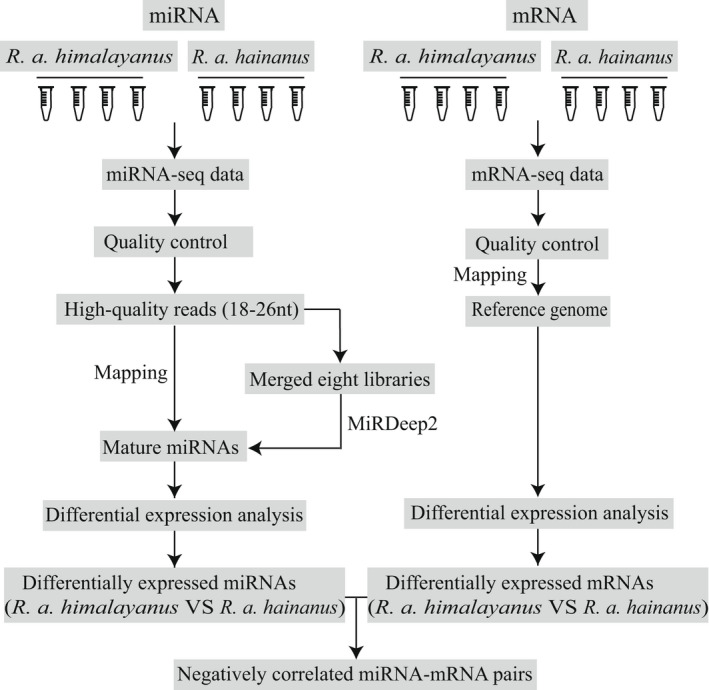
The pipeline of miRNAs and mRNAs bioinformatic analysis

We used the following pipeline to identify miRNAs (Figure 2). Raw small RNA sequencing data were processed using Cutadapt v2.6 (Martin, 2011) by trimming adapter sequences and removing reads with unknown bases. Low‐quality reads were further filtered out using TRIMMOMATIC version 0.38 (Bolger et al., 2014) with parameters of SLIDINGWINDOW: 4:20. Because miRNAs are typically ~22 nucleotides (nts), only reads of 18–26 nts were retained in the following analysis.

We used miRDeep2.0.0.8 (Friedländer et al., 2012) to predict precursor and mature miRNAs. Specifically, trimmed reads from all eight samples were combined and mapped to the reference genome of *Rhinolophus affinis* using the Mapper.pl, a module of miRDeep2. This reference genome contains 587 scaffolds with N50 of 30.08 Mb, 20,180 annotated genes and BUSCO score of 96.9% (unpublished data from G Li), whose quality is comparable to recently published high‐quality genomes of bats (Jebb et al., 2020). We used a set of known mature miRNAs including those identified in miRBase v22 (Kozomara & Griffiths‐Jones, 2014) and those published in three other bat species (539 in *Myotis myotis*, Huang et al., 2016; 404 in *Myotis lucifugus*, Biggar & Storey, 2014; 196 in *Myotis ricketti*, Yuan et al., 2015; See Dryad file T2). We retained all known and novel miRNAs with a minimum miRDeep2 score of 10 and minimum read depths of 5. Following Jebb et al. (2020), we manually moved those novel miRNAs sharing the same seed region with a known miRNA to the known category. The final novel miRNAs in this study mean those that have not been reported in any organisms until now.

### 2.4 Identification of specific miRNAs in echolocating mammals

**FIGURE 3:**
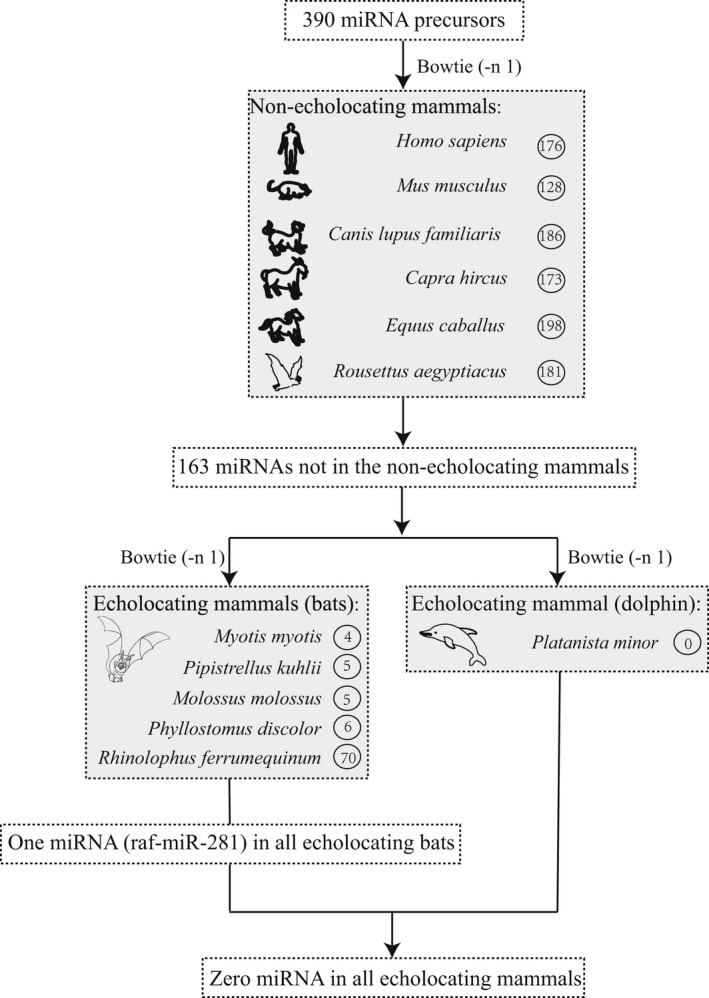
The pipeline of identification of candidate miRNAs putatively associated with echolocation. The number in the circle represents the number of miRNAs occurring in the genome of species

This is the first study to characterize miRNAs in cochlea from an echolocating mammal. To identify candidate miRNAs specific to echolocating mammals, we searched all miRNAs identified here in genomes of other echolocating and nonecholocating mammals (Figure 3). First, all miRNA precursors were mapped to genomes of one nonecholocating bat (*Rousettus aegyptiacus*) and five other nonecholocating mammals (human, mouse, dog, goat, and horse). Second, unmapped miRNA precursors were aligned to recently published high‐quality genomes of five echolocating bats (*Rhinolophus ferrumequinum*, *Phyllostomus discolor*, *Myotis myotis*, *Pipistrellus kuhlii*, and *Molossus molossus*) (Jebb et al., 2020) and one echolocating dolphin (*Platanista minor*). All these genomes were downloaded from NCBI (National Center for Biotechnology Information). Following Jebb et al. (2020), we used Bowtie (v1.0.0) (Langmead et al., 2009) with the ‐n 1 parameter. We assume that those miRNAs occurring in all echolocating bats or all echolocating mammals (echolocating bats and dolphin) but absent in nonecholocating mammals are candidates putatively associated with the evolution of echolocation.

### 2.5 miRNA differential expression analysis

**FIGURE 4:**
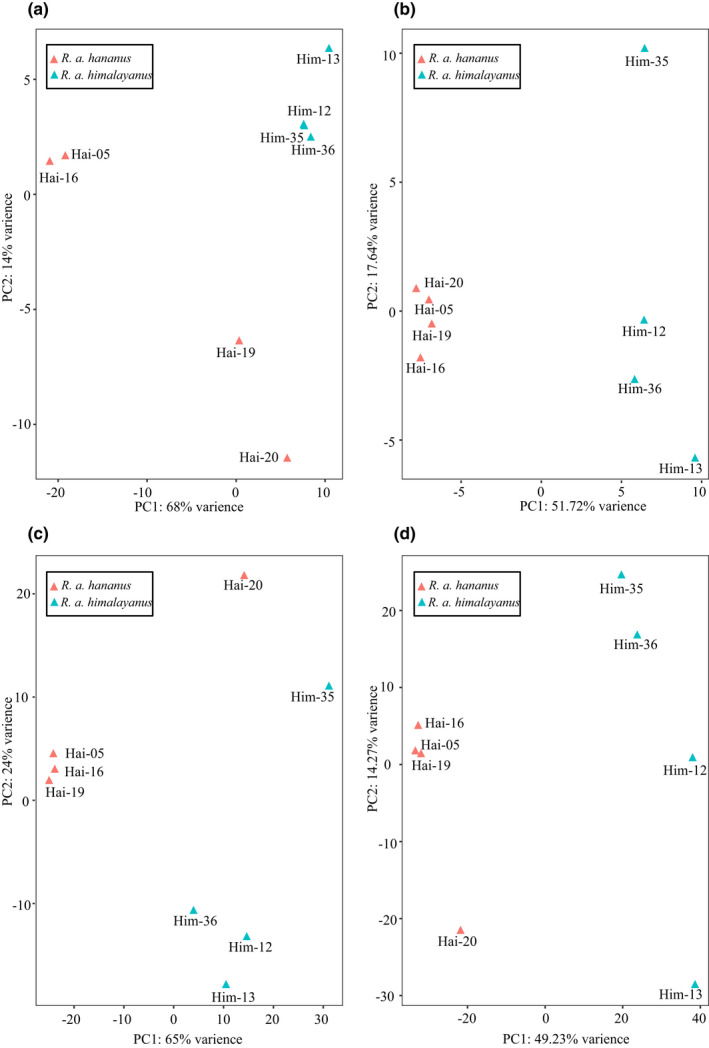
Principal component analysis (PCA) showing overall miRNA and mRNA expression patterns in cochlea among all eight individuals of *R. a. Himalayanus* and *R. a. hainanus*. (a) and (c) based on raw counts matrix of miRNA and mRNA, respectively; (b) and (d) based on the SVA‐adjusted expression matrix of miRNA and mRNA, respectively

To perform differential expression analysis (Figure 2), trimmed small RNA reads from each sample were mapped to the mature and precursor miRNAs identified in *R. affinis* above using the script quantifier.pl module in miRDeep2 package to quantify expression levels of miRNAs. Mapped read counts in each sample were then normalized using DESeq function in DESeq2 v1.30.1. (Love et al., 2014) in order for comparison across samples. Principal component analysis (PCA) revealed a batch effect in our current data (Figure 4a), possibly because samples were sequenced in two different lanes. After removal of batch effect using R package SVA (Leek et al., 2012) with surrogate variables of one, PCA based on normalized miRNA expression matrix clearly separated all samples into two clusters, corresponding to each of the two subspecies (Figure 4b). Prior to differential expression analysis, we filtered out the lowly expressed miRNAs with the average CPM < 1 among all samples. Then, differentially expressed miRNAs between the two subspecies were determined using both DESeq2 and edgeR (Robinson et al., 2010) with significant results (Benjamini & Hochberg, 1995, padj < 0.05 in DESeq2 and FDR < 0.05 in edgeR) and |log_2_(fold change)| > 1. To test whether different amounts of sequencing data generated for each sample affected the current results, we repeated the differential expression analysis based on similar amounts of data for each sample. For this, we reduced sequencing data of each sample to 4.6 million reads (the lowest amount of data, Dryad file T3).

### 2.6 mRNA differential expression analysis

We used the following pipeline to analysis mRNA‐seq data (Figure 2). Specifically, raw reads from mRNA‐seq were processed using TRIMMOMATIC v0.38 (Bolger et al., 2014) with the parameters of SLIDINGWINDOW:4:20. For each sample, filtered reads were mapped to the reference genome of *R. affinis* using HISAT2 v2.2.0 (Kim et al., 2015) with default setting. SAMtools v1.11 (Li et al., 2009) was used to generate sorted BAM files. Mapped reads in the alignment were quantified using FeatureCounts v2.0.1 (Liao et al., 2014) and read count matrix across samples was normalized in DESeq2. Similar to miRNA data above, batch effect was detected in the mRNA data with PCA (Figure 4c) which was removed using svaseq function with surrogate variables of 1. After removal of batch effect, normalized mRNA expression matrix of all samples clearly clustered by subspecies (Figure 4d). Then, we filtered out those lowly expressed transcripts with the average CPM < 1 among all samples. Differentially expressed genes (DEGs) between the two subspecies were also identified using both DESeq2 and edgeR with the same criteria as in the miRNA above.

### 2.7 MiRNA target prediction

To assemble transcripts expressed in cochlea, we applied a reference‐guided assembly method using StringTie v2.1.5 (Pertea et al., 2015) with *R. affinis* genome as the reference. Filtered RNA‐seq data from all eight samples (see below mRNA differential expression analysis) were combined together and mapped to the *R. affinis* genome using TopHat v2.1.1 (Trapnell et al., 2009) with default parameters. Assembled transcripts by StringTie were extracted with the *gffread* module in the cufflinks v2.2.1 (Trapnell et al., 2012). Then, the transcripts were annotated by searching against the coding sequences (CDSs) of 20,180 genes retrieved from the *R. affinis* genome using BLASTN with e‐value 10^−6^, sequence identify >90%, and alignment length > 100 nt. Sequences downstream of the end of the alignment were extracted from the transcript of each gene as 3′UTRs. We only kept those 3′UTRs with the length of 25–2500 nts, resulting in a total of 10,212 3′UTRs used for the prediction of miRNA target sites.

We predicted target sites of differentially expressed miRNAs at 3′UTRs using miRanda v3.3a (Enright et al., 2003) and TargetScan v 7.0.2 (Agarwal et al., 2015). In order to obtain more reliable results, we only retained those miRNA‐mRNA interactions predicted by both types of software. For miRanda, the entire miRNAs sequences were used as the input and the following parameters were applied: with energy threshold −10 and strict 5′seed pairing. For TargetScan, the seed region (2–8) of miRNAs was extracted and used as the input to predict the miRNA targets with default parameters. We used the conserved seeds for target prediction and retained prediction type of 7mer‐1a, 7mer‐m8, and 8mer.

### 2.8 Identification of differentially expressed miRNA targets

With the lists of differentially expressed miRNA and their differentially expressed target genes, we manually extracted miRNA‐gene pairs with positively and negatively correlated expression level. As an alternative method, we used Pearson function in MIRLAB (Le et al., 2015) package to test for Pearson correlations of expression level between differentially expressed miRNAs and their differentially expressed target genes. This analysis returns a matrix with the correlation coefficients/scores from −1 to 1, indicating negative (from −1 to 0) and positive (from 0 to 1) miRNA‐gene pairs. To reduce the false positives, we only retained the miRNA‐gene pairs identified by both methods.

### 2.9 Functional enrichment analysis

We used Metascape (http://metascape.org) to perform Gene Ontology (GO) enrichment analysis with the Custom Analysis module (Zhou et al., 2019). A total of 13,409 expressed genes in cochlea were included as the background list. Significantly enriched GO terms were identified using q‐value <0.05 adjusted for multiple tests in the Benjamini–Hochberg procedure (Hochberg & Benjamini, 1990). We conducted GO analyses on target genes in negatively correlated miRNA–gene pairs. Redundancy of the GO terms were reduced using the REVIGO clustering algorithm (http://revigo.irb.hr/). We then used the scatterplots to visualize the clustered GO terms based on their semantic similarities.

In addition, we used a candidate gene approach to identify loci associated with echolocation call frequency variation following Sun et al. (2020). Specifically, we compared the target genes to the list of genes that are known to cause hearing loss and/or deafness in human or mouse (called hearing genes). We collected such 652 genes from MGI (Mouse Genome Informatics), 121 genes from HHLH (Hereditary Hearing Loss Homepage. https://hereditaryhearingloss.org) and 166 genes causing increased or absent threshold for auditory brainstem response (ABR) from IMPC (The International Mouse Phenotyping Consortium, MP:0011967).

## 3 RESULTS

### 3.1 Identification of miRNAs in *R. affinis*

**FIGURE 5:**
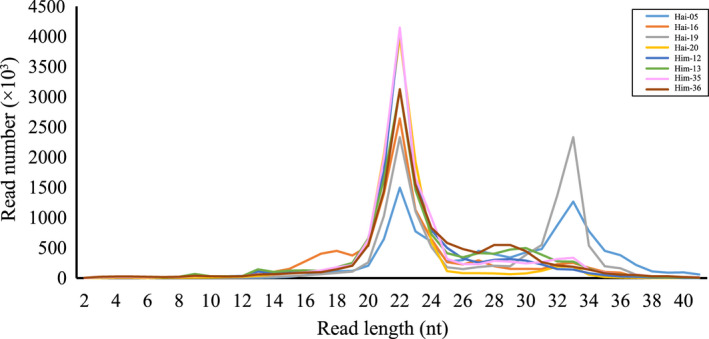
Length distribution of sequenced miRNAs for each sample. Sample information has been described in Table 1

To annotate miRNAs genes in the *R. affinis* genome, we used small RNA‐seq reads of the cochlea tissue from the eight samples (two subspecies, four samples per subspecies). After removing adaptors and low‐quality reads, we obtained an average of 12,160,468 reads per sample with the length of 18–35 nts (Dryad file T3 and Figure 5). The length distribution of the reads exhibited a peak at 22 nts for all samples while two samples (Hai‐05 and Hai‐19) had another peak at 33 nts (Figure 5). Reads of the second peak in Hai‐05 and Hai‐19 may belong to PIWI‐interacting RNAs (see also Ord et al., 2020). Two peaks shown in the length distribution of small RNA reads have also been documented in other studies (e.g., Fu et al., 2020; Luo et al., 2021). Because miRNAs typically have ~22 nts in length, we only retained reads of 18–26 nts, resulting in an average of 8,266,875 reads per sample (Dryad file T3).

With small RNA‐seq reads of the eight samples and the reference genome of *R. affinis*, the miRDeep2 predicted 390 miRNAs precursors. After removing redundancy, 365 unique mature miRNAs were retained, including 244 known and 121 novel miRNAs (sequence of all mature miRNAs and their corresponding precursors has been provided in Dryad file T4).

### 3.2 One specific miRNA was identified in all echolocating bats

Following Jebb et al. (2020), we used Bowtie with the ‐n 1 parameter to test for the miRNA genes existing in genomes of all echolocating mammals but absent in genomes of nonecholocating mammals. For all 390 miRNA precursors, 10 were present in at least two echolocating bats but absent in nonecholocating mammals (Table 2). More importantly, we found that one miRNA (raf‐miR‐281) was shared by all echolocating bats (Table 2). A total of 435 target genes were predicted by this miRNA and 16 of them were in the list of hearing genes (Dryad file T5). However, no miRNA was shared by all echolocating mammals (bats and dolphin).

**TABLE 2:** Ten miRNAs shared in at least two echolocating bats with reference quality genomes (Jebb et al., 2020). Yes and no means presence and absence in the genome of species, respectively. MiRNA shared in all echolocating bats was shown in bold

MiRNAs/species	*Rhinolophus ferrumequinum*	*Molossus molossus*	*Myotis myotis*	*Phyllostomus discolor*	*Pipistrellus kuhlii*
raf‐miR‐87	Yes	No	Yes	Yes	No
raf‐miR‐89	Yes	Yes	Yes	No	Yes
raf‐miR‐162	Yes	No	No	No	Yes
raf‐miR‐280	Yes	Yes	No	No	No
**raf‐miR‐281**	**Yes**	**Yes**	**Yes**	**Yes**	**Yes**
raf‐miR‐314	Yes	No	No	Yes	No
raf‐miR‐317	Yes	Yes	No	Yes	No
raf‐miR‐325	Yes	No	No	Yes	Yes
raf‐miR‐331	Yes	No	No	Yes	No
raf‐miR‐351	Yes	Yes	No	No	No

### 3.3 Identification of differentially expressed miRNAs

In this study, we conducted differential expression analysis on cochlea miRNA‐seq data from two subspecies of *R. affinis* (*himalayanus* and *hainanus*), which show over 15 kHz difference of echolocation call frequency (Table 1). Using both DESeq2 and edgeR, we identified 48 differentially expressed miRNAs (DEMs) between the two subspecies (Dryad file T6). To test whether the relative low amount of sequencing reads of Hai‐05 and Hai‐19 comparing with other samples (Dryad file T3) affected the current results, we repeated the differential expression analysis by reducing the sequencing data of each sample to the one in Hai‐05, which resulted in 50 DEMs. Almost all of these DEMs (45 of 50) were overlapped with the ones based on the whole data above (Dryad file T7), indicating that the retained sequencing reads for each sample were enough to produce reliable results.

We predicted the target genes of all 48 DEMs using two commonly used programs (miRanda and TargetScan). A total of 7741 target genes and 27,067 miRNA–gene relationship pairs were predicted by both programs. Similar to the results in Franchini et al. (2019), almost all retained miRNA‐gene pairs were from the predictions of miRanda (98.4%, Figure S5).

### 3.4 Identification of differentially expressed genes

In this study, we generated the matched mRNA‐seq data for each sample with an average of 20,976,159 read pairs 150 bp long per sample, 96.88% of which were retained after quality control (Dryad file T3). A total of 1766 DEGs were identified between the two subspecies using both DESeq2 and edgeR (Dryad file T8). To obtain the Ensembl Gene ID of each DEG, we performed BLAST searches against nt (Nucleotide Sequence Database) and nr (Non‐Redundant Protein Sequence Database) with the coding sequences of these DEGs. A total of 1418 DEGs have Ensembl Gene IDs and 1015 of them have 3′UTRs. Among them, 44 were found to be in the list of hearing genes (Dryad file T8). It is to be noted that only 135 DEGs identified in this study were also shown in the list of 799 DEGs identified between the same two subspecies in Sun et al. (2020). Functional enrichment analysis on these 135 overlapped genes revealed zero significant GO terms.

### 3.5 Identification of differentially expressed miRNA targets

**FIGURE 6:**
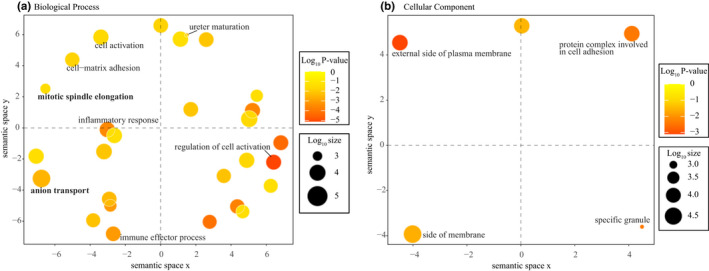
Multidimensional scaling plots showing significant gene ontology (GO) terms enriched on target DEGs regulated negatively by miRNAs. Clustering was conducted according to the semantic similarity of GO terms. The color and size of circles represent the p‐value calculated by Metascape and the frequency of the GO term in GO annotation database, respectively. The highly shared GO terms were displayed with their descriptions. (a) Clustering of the Biological Processes GO terms. (b) Clustering of the Molecular Function GO terms

It is to be noted that as stated above, not all DEGs were annotated with 3′UTRs and those DEGs without 3′UTRs would be missed in the expression correlation analysis with DEMs. Among the 27,067 miRNA–gene pairs, we first retained the pairs involving DEGs (745 target DEGs, 2501 miRNA–gene pairs) (Dryad file T9). We further examined miRNA–gene expression correlation and identified 1084 negatively correlated miRNA–gene pairs (555 target DEGs) and 1272 positively correlated miRNA‐gene pairs (575 target DEGs) by two different approaches. Because miRNAs usually negatively regulate mRNAs by inhibiting their production, here we only focused on negatively correlated miRNA‐gene pairs. Among the 555 target DEGs regulated negatively by miRNAs, 19 were in the list of hearing genes (Dryad file T10). A further GO enrichment analysis on these target DEGs revealed 74 significant GO terms and a majority of GO terms were found to be associated with immunity (Dryad file T11 and Figure 6).

## 4 DISCUSSION

As far as we know, this is the first study to characterize miRNAs of the cochlea in an echolocating mammal. The number of mature miRNAs (365) identified here was either more or less than that in other bats (e.g., 468 in *Myotis myotis*, Huang et al., 2016 and 404 in *Myotis lucifugus*, Biggar & Storey, 2014; 196 in *Myotis ricketti*, Yuan et al., 2015 and 217 in *Eptesicus fuscus*, Platt et al., 2014). This contrast of the number of miRNAs across studies might result from different tissues used because miRNAs have been shown to be expressed with tissue specificity (Lagos‐Quintana et al., 2002; Xu et al., 2007). In addition, expression of miRNAs also depends on ecological or environmental context, for example, of torpid and active states in hibernating mammals (Biggar & Storey, 2014, 2018; Yuan et al., 2015).

The current miRNA‐seq data from the cochlea tissue of an echolocating bat (*R. affinis*) allow us to investigate whether miRNAs play an important role in the evolution of echolocation. We found 10 miRNAs present in at least two echolocating bats but not in other nonecholocating mammals. Interestingly, one of them (raf‐miR‐281) was shared by all echolocating bats and targeted genes of this miRNA included several hearing genes, which have been proved to be important in echolocation of bats (e.g., *KCNQ4*, Liu et al., 2012). Therefore, raf‐miR‐281 might be a candidate miRNA that is essential in the evolution of echolocation in bats, which can be tested by functional tests of this miRNA and its targeted genes in the future. However, this candidate miRNA was not present in other echolocating mammals (e.g., dolphin), suggesting independent origins of echolocation in bats and dolphin.

Our current results also support an important role of miRNAs in echolocation call frequency variation between the two subspecies. First, we found that almost half of known hearing genes in the list of all DEGs were regulated negatively by differentially expressed miRNAs (DEMs). Second, target DEGs regulated by DEMs included multiple important hearing genes (*Piezo1*, *Piezo2*, and *CDH23*). PIEZO (PIEZO1/2) has been identified as vertebrate mechanically gated ion channels (Coste et al., 2012) and may play an essential role in mechnotransduction in auditory hair cells (Beurg & Fettiplace, 2017). A recent study has shown that PIEZO2 mediates ultrasonic hearing during social behaviors in mice (Li et al., 2021). In contrast to *Piezo2* (Beurg & Fettiplace, 2017; Li et al., 2021; Schneider et al., 2019; Wu et al., 2017; Zheng et al., 2019), less is known about the role of *Piezo1* in mechanosensory cells. In this study, *Piezo1* and *Piezo2* were regulated by two different miRNAs (raf‐miR‐278 for *Piezo1* and raf‐miR‐207 for *Piezo2* (Dryad file T10). In the future, functional assays can be conducted to test for the interactions of these two miRNAs and *Piezo1/2* and more importantly to assess their roles in ultrasonic hearing of echolocating mammals. Another one, *CDH23*, has been shown to be important in the ultrasonic hearing of echolocating mammals (e.g., *Cdh23*, Shen et al., 2012).

Although it is not one of our main aims, we also compared our results of mRNA differential expression analysis to a previous study that used the same two subspecies as the system (Sun et al., 2020). To our surprise, we found a low number of shared DEGs between the current study and Sun et al. (2020). This low level of consistency might result from: (1) different individuals sampled; (2) different sampling times; (3) different references used in reads mapping (a high‐quality reference genome in this study while a de novo assembled transcriptome in Sun et al., 2020); (4) the high degree of heterogeneity of cell types in the cochlea. Nevertheless, the shared DEGs between these two studies could be considered as the most candidates related to echolocation call frequency variation between the two subspecies.

## 5 CONCLUSION

In this study, we characterized miRNAs of the cochlea in an echolocating bat. By searching these miRNAs in high‐quality genomes of other echolocating and nonecholocating mammals, we found one specific miRNA in all echolocating bats that might be essential in the evolution of echolocation in bats. In addition, our current study adds to a growing number of works that support an important role of miRNAs in phenotypic diversifications of wild animals (e.g., Franchini et al., 2016, 2019).

## AUTHOR CONTRIBUTIONS

**Qianqian Li:** Formal analysis (lead); writing – original draft (equal). **Xiuguang Mao:** Conceptualization (lead); writing – original draft (lead). **Wenli Chen:** Data curation (supporting).

## CONFLICT OF INTEREST

The authors declared that they have no competing interests.

## Data Availability

Sequencing reads from this article have been deposited to NCBI's Sequence Read Archive database (SRA) under BioProject accession no. PRJNA764560. All supplementary information files are available from the Dryad Repository https://doi.org/10.5061/dryad.wh70rxwqm.
